# IgM nephropathy: timely response to a call for action

**DOI:** 10.12861/jrip.2014.03

**Published:** 2013-11-02

**Authors:** Muhammed Mubarak, Hamid Nasri

**Affiliations:** ^1^Histopathology Department, Sindh Institute of Urology and Transplantation, Karachi, Pakistan; ^2^Department of Nephrology, Division of Nephropathology, Isfahan University of Medical Sciences, Isfahan, Iran

**Keywords:** Complement, Focal segmental glomerulosclerosis, Immunoglobulin M, Nephropathy, Proteinuria

## Abstract

Immunoglobulin M (IgM) is the largest antibody molecule and is deposited in the glomeruli in a wide variety of both primary and secondary glomerulopathies. The data on its pathogenic role are conflicting till date. A recent study provides evidence for the involvement of natural antibody IgM in fixing and activating complement and causing glomerular injury, proteinuria, and glomerulosclerosis in an animal model. This finding is important in understanding the pathogenesis of the related disorder of IgM nephropathy.

**Implication for health policy/practice/research/medical education:**

IgM nephropathy (IgMN) is a controversial clinicoimmunopathologic entity in the arena of primary glomerulopathies. Recently, many clinicopathological studies have been published, but there is severe lack of information on etiopathogenesis of the condition. A recent study has found that naturally occurring IgM antibody is pathogenic and plays a role in causing damage in animal models and focal segmental glomerulosclerosis (FSGS). This study provides an important stimulus for basic research focused on elucidating the pathophysiology of IgMN.

Strassheim *et al*. (1) provide fairly strong evidence in an animal model for the pathogenic role of IgM in inducing glomerular injury and ultimately the glomerulosclerosis. They have demonstrated that naturally occurring IgM antibody binds to neo-epitopes in the glomeruli exposed after some environmental insult and that this IgM then binds and activates the complement proteins, further aggravating the glomerular injury. It is worth mentioning here that IgM is the most common immunoreactant found on immunoflourescence (IF) microscopy of renal biopsies in a wide variety of both primary and secondary glomerulopathies (1). But, it was uncertain till present, whether the glomerular deposition of this antibody is pathogenically important or it just represents a nonspecific trapping of the molecule in an already injured glomerulus. This uncertainty was all the more important in relation to the primary glomerulopathy of IgM nephropathy (IgMN), a largely controversial entity till date (2-4). The findings of the study by Strassheim *et al*. (1) may prove to be a landmark achievement in clarifying the nosologic status of IgMN, a disease characterized by predominant IgM deposits. The authors have shown that IgM deposition precedes the development of glomerulosclerosis in adriamycin induced nephropathy model in mice, thus precluding the nonspecific trapping theory. The authors have also elegantly demonstrated the colocalization of IgM and activation products of complement proteins such as C4d and C3d, further supporting the role of antibody in activating complement cascade. Furthermore, strategies to reduce natural antibody IgM in the study resulted in reduction in glomerular injury, immune reactants depositions and proteinuria. All these findings support a pathogenic role of IgM antibodies in glomerulopathies. However, this may not be involved in all glomerular diseases. To prove this point, the authors also reviewed the immunohistochemical (IHC) findings of 174 cases of focal segmental glomerulosclerosis (FSGS) in humans and found sole IgM deposition in 23% of cases, IgM and C3 in 7% and C3 alone in 2% cases. Overall, the deposition of IgM and/or C3 was detected in approximately one third of cases. It should be noted that this review involved the retrospective analysis of IHC finding of the reports. The authors stated that the percentage of IgM and/or C3 positivity may be higher if studied carefully and prospectively. This finding provides evidence that IgM deposition plays a role in potentiating glomerular injury in only a subset of patients with FSGS. It is well known that FSGS is not a specific disease, but a histologic pattern and harbinger of progressive damage, found in a wide variety of both primary and secondary glomerular diseases (5).

Although IgMN was first described way back in 1970s, interest in the disease soon faded and many investigators did not recognize it as a separate entity (6-8). More recently, renewed interest has been generated about the disease and many clinicopathological studies have attempted to characterize the presenting features, morphological findings and the prognostic factors of IgMN in both children and adults (9-15). But research directed toward elucidating the mechanism of the disease has clearly lagged behind, mainly due to the apathy of researchers from the developed countries. We have earlier stressed the need for more basic research into the disease (16). This study may be considered a timely response to that call and will help open the avenues for further basic research aimed at identifying the pathogenetic mechanisms of the disease. It also remains to be proven whether IgM can deposit on its own and initiate glomerular injury or it only does so after some other glomerular insult.

In conclusion, the study by Strassheim *et al*. represents a significant development in the area of basic research into the pathogenic role of natural antibody IgM in inducing glomerular damage and glomerulosclerosis. Further studies in animal models and humans are needed to fully unravel the pathogenicity of the antibody in other glomerular diseases, especially the IgMN.

## Authors’ contributions

All authors wrote the manuscript equally.

## Conflict of interests

The authors declared no competing interests.

## Ethical considerations

Ethical issues (including plagiarism, data fabrication, double publication) have been completely observed by the author.

## Funding/Support

None.
